# Early discordance between alpha-fetoprotein response and albumin-bilirubin deterioration refines response assessment in unresectable hepatocellular carcinoma receiving first-line immune-combination therapy: a real-world landmark cohort study

**DOI:** 10.3389/fonc.2026.1954746

**Published:** 2026-10-19

**Authors:** Haitao Wu, Pengju Tang, Jinbo Gong, Xintao Shi, Yiming Zhao, Zhong Chen

**Affiliations:** Department of Hepatobiliary and Pancreatic Surgery, Affiliated Hospital of Nantong University, Nantong University Medical School, Nantong, China

**Keywords:** albumin-bilirubin score, alpha-fetoprotein, hepatocellular carcinoma, immune-combination therapy, landmark analysis, liver reserve, response assessment

## Abstract

**Background:**

Early assessment during first-line immune-combination therapy for unresectable hepatocellular carcinoma (HCC) must consider both antitumor activity and hepatic tolerance. Existing integrated approaches have paired early AFP variation with baseline liver reserve, but they do not directly ask whether hepatic reserve is preserved or deteriorates over the same early treatment interval. We investigated whether early alpha-fetoprotein (AFP) response and change in modified albumin-bilirubin (ALBI) grade define clinically interpretable response-tolerance phenotypes.

**Methods:**

This retrospective landmark cohort included AFP-evaluable patients with unresectable HCC who received first-line immune-combination therapy. Follow-up for the primary analysis began 70 days after treatment initiation. AFP response was defined as a landmark-to-baseline ratio of 0.80 or lower among patients with baseline AFP of at least 20 ng/mL; modified ALBI deterioration was defined as worsening by at least one category. Patients were classified as concordant favorable, response-liver vulnerability, nonresponse-liver preserved, or concordant failure. The primary endpoint was progression-free survival (PFS).

**Results:**

Of 286 treated patients, 68 (23.8%) had baseline AFP below 20 ng/mL and were not AFP-evaluable; 166 patients entered the primary day-70 cohort. The four phenotypes included 58, 36, 43, and 29 patients, respectively. Relative to concordant favorable, concordant failure had the strongest adjusted association with shorter PFS (hazard ratio [HR] 2.85, 95% confidence interval [CI] 1.63–4.99; P <0.001), followed by nonresponse-liver preserved (HR 1.69, 95% CI 1.02–2.79; P = 0.040). The estimate for response-liver vulnerability was smaller and imprecise (HR 1.49, 95% CI 0.89–2.49; P = 0.130). The combined non-favorable group was associated with shorter PFS (HR 1.79, 95% CI 1.18–2.72; P = 0.006). The hepatic-decompensation estimate was imprecise after parsimonious adjustment (odds ratio 2.42, 95% CI 0.90–6.50; P = 0.081). Overall survival (OS) estimates were also imprecise; the largest point estimate was observed for concordant failure (HR 2.09, 95% CI 1.00–4.38; P = 0.050).

**Conclusion:**

Early AFP-ALBI discordance distinguished different patterns of tumor-marker response and hepatic tolerance during immune-combination therapy. In AFP-evaluable patients with an ambiguous first assessment, a non-favorable biochemical pattern may support earlier multimodal reassessment of tumor status, liver function, and treatment feasibility, but it should not be used as a stand-alone rule for changing therapy.

## 1 Introduction

Hepatocellular carcinoma (HCC) remains a major cause of cancer-related death, and its global burden is projected to increase as populations age and chronic liver disease persists (1–3). In contrast to most solid tumors, prognosis and treatment feasibility in HCC depend on both tumor extent and the functional reserve of the underlying liver. The Barcelona Clinic Liver Cancer framework and current American Association for the Study of Liver Diseases (AASLD) and European Association for the Study of the Liver (EASL) guidance therefore consider tumor burden, performance status, liver function, and treatment setting together rather than relying on anatomical stage alone (4–6).

First-line treatment for unresectable HCC has evolved rapidly. Atezolizumab plus bevacizumab and durvalumab plus tremelimumab established immune-combination therapy as standard options, while sintilimab plus a bevacizumab biosimilar and camrelizumab plus rivoceranib extended the phase 3 evidence in predominantly Asian populations (7–11). Immune checkpoint inhibitor (ICI)-based combinations with tyrosine kinase inhibitors (TKIs) are also used in routine practice and remain under clinical evaluation (12, 13). Because these regimens differ in antitumor activity, vascular effects, and hepatic toxicity, early treatment assessment has become increasingly important and increasingly complex.

Imaging remains central to response assessment. RECIST 1.1 provides a standardized size-based approach, whereas modified RECIST (mRECIST) evaluates viable enhancing tumor and is particularly relevant to HCC (14–16). Even so, the first post-treatment scan frequently shows stable disease, a broad category that may represent durable control, emerging progression, or treatment-related change. Routinely measured biochemical markers could help resolve this uncertainty, provided that they are interpreted alongside, rather than in place of, imaging.

Alpha-fetoprotein (AFP) is informative only in AFP-producing HCC, but it remains the most widely available circulating marker of tumor activity. Early declines in AFP have been associated with radiographic response and survival during ICI-based treatment and atezolizumab-bevacizumab therapy (17–22). A meta-analysis also supported the prognostic value of early AFP response and identified a decline of >20% within the first several weeks as a practical threshold (23). AFP kinetics alone, however, cannot distinguish ineffective tumor control from a treatment course complicated by loss of hepatic reserve.

The albumin-bilirubin (ALBI) score offers an objective measure of liver function and has been validated across HCC stages and treatment settings (24–26). Modified ALBI categories provide finer discrimination within the intermediate range of liver function and may detect clinically relevant deterioration that is not apparent from the broader Child–Pugh classes (27, 28). We therefore considered AFP response and modified ALBI deterioration as complementary dimensions of early treatment assessment. A day-70 landmark and additional time-origin sensitivity analyses ensured that phenotype assignment preceded outcome follow-up (29–31). We examined whether concordant and discordant AFP-ALBI patterns were associated with subsequent outcomes after first-line immune-combination therapy and whether they added clinically interpretable information to imaging without being treated as a rule for changing therapy. Rather than combining an on-treatment AFP change with baseline liver reserve alone, the present analysis asked whether hepatic reserve itself was preserved or deteriorated over the same early interval, with subsequent outcome follow-up beginning only after the joint state was established.

## 2 Materials and methods

### 2.1 Study design and ethics

This single-center retrospective cohort study included patients with unresectable HCC who received first-line immune-combination therapy in routine practice at the Affiliated Hospital of Nantong University. The report was prepared in accordance with the STROBE recommendations for observational studies (32). The study conformed to the Declaration of Helsinki and was approved by the Ethics Committee of the Affiliated Hospital of Nantong University (approval no. NTU022396). No identifiable patient information is presented.

### 2.2 Study population and treatment context

HCC was diagnosed histologically or according to accepted clinicoradiographic criteria. Eligible first-line regimens combined an immune checkpoint inhibitor with an anti-VEGF antibody, a TKI, another immune checkpoint inhibitor, or another systemic partner used in routine care. Patients who had previously received systemic therapy for HCC were excluded from the first-line cohort. Eligibility for analysis required a documented treatment start date, baseline AFP and liver-function measurements, and sufficient follow-up to establish the applicable landmark and outcomes. Systemic regimen class was reconstructed from the systemic regimen itself and analyzed independently of local therapy delivered during the first 8 weeks.

### 2.3 Landmark framework and analysis populations

Because AFP response and ALBI deterioration are measured after treatment begins, the primary survival analysis used a fixed day-70 landmark. This approach prevented outcome follow-up from starting before phenotype status could be determined (29–31). The primary cohort included patients with baseline AFP of at least 20 ng/mL, complete AFP, albumin, and total bilirubin measurements obtained during days 28–70, and no progression, death, or loss to follow-up on or before day 70. Follow-up for PFS and Overall survival (OS) began on day 70. Day 70 was defined as 70 calendar days after the recorded treatment start date. When more than one complete AFP, albumin, and bilirubin assessment was available within days 28–70, the complete assessment closest in calendar time to day 70 was used for phenotype assignment.

First imaging response was described among patients in the primary cohort with evaluable post-treatment imaging. For survival analyses restricted to imaging-defined stable disease, the joint time origin was the later of day 70 or the date of the first post-treatment scan; patients were required to be alive, progression-free, and under follow-up through that joint landmark. RECIST 1.1 and mRECIST stable-disease cohorts were analyzed as exploratory clinical scenarios.

### 2.4 AFP-ALBI response-tolerance phenotypes

AFP response was defined as a landmark-to-baseline AFP ratio of 0.80 or lower, equivalent to a decline of at least 20%, among patients with baseline AFP of at least 20 ng/mL. The ALBI score was calculated as 0.66 × log10(total bilirubin in micromoles per liter) − 0.085 × albumin in grams per liter (24). Modified ALBI categories were grade 1 (ALBI of −2.60 or lower), grade 2a (greater than −2.60 to −2.27), grade 2b (greater than −2.27 to −1.39), and grade 3 (greater than −1.39) (28). Modified ALBI deterioration was defined as worsening by at least one category between baseline and the early assessment. A categorical modified-ALBI definition was chosen because it maps the continuous score to clinically familiar liver-reserve strata and makes concordant and discordant states directly interpretable; continuous ALBI change was examined separately to assess sensitivity to category boundaries.

Patients were assigned to one of four phenotypes: concordant favorable (AFP response without modified ALBI deterioration), response-liver vulnerability (AFP response with modified ALBI deterioration), nonresponse-liver preserved (no AFP response and no modified ALBI deterioration), or concordant failure (no AFP response with modified ALBI deterioration). For selected secondary, sensitivity, and subgroup analyses, the three phenotypes other than concordant favorable were combined as a non-favorable group.

### 2.5 Imaging assessment

The first post-treatment scan was classified as complete response, partial response, stable disease, or progressive disease according to RECIST 1.1 and mRECIST (14, 15). Paired RECIST assessments from two readers were available for the agreement analysis. Agreement was described using the complete cross-classification matrix, observed agreement, and unweighted Cohen’s kappa with a bootstrap confidence interval.

### 2.6 Outcomes

The primary outcome was progression-free survival (PFS), defined from the day-70 landmark to radiographic or clinical progression, death from any cause, or censoring at the last documented follow-up. OS was defined from day 70 to death from any cause or censoring. Secondary post-landmark outcomes were progression or death within 90 days after the day-70 landmark, treatment discontinuation within 90 days after the day-70 landmark, and hepatic decompensation within 3 months after the day-70 landmark. Hepatic decompensation was adjudicated from clinical records on the basis of decompensating events including new or worsening ascites, hepatic encephalopathy, or variceal bleeding. Grade 3 or higher adverse events were captured over the entire treatment follow-up and were treated as a descriptive safety outcome rather than a post-landmark endpoint. Analyses restricted to imaging-defined stable disease began at the joint phenotype-imaging landmark.

### 2.7 Statistical analysis

Continuous variables are reported as medians with interquartile ranges, and categorical variables as n/N (%). Descriptive comparisons across phenotypes used nonparametric or categorical tests, as appropriate. These P values were not interpreted as evidence of balance between non-randomized groups. Exposure-defining AFP, albumin, and bilirubin values were not imputed.

Kaplan–Meier methods were used to display PFS and OS. Cox proportional hazards models estimated hazard ratios (HRs) with 95% confidence intervals (CIs), with concordant favorable as the reference phenotype. The primary adjusted PFS model included phenotype, Eastern Cooperative Oncology Group (ECOG) performance status (0–2, modeled ordinally), macrovascular invasion, extrahepatic metastasis, tumor burden score, baseline continuous ALBI score, and systemic regimen class (10 regression parameters for 130 PFS events; 13.0 events per parameter). The reduced OS model included phenotype, ECOG performance status, macrovascular invasion, and baseline continuous ALBI score (six regression parameters for 64 deaths; 10.7 events per parameter). The covariate sets were chosen on clinical grounds and were not selected from univariable P values. Post-landmark secondary binary outcomes were examined using parsimonious logistic models containing the combined non-favorable exposure and baseline continuous ALBI score.

Sensitivity analyses used a laboratory window restricted to days 28–42, the actual laboratory date as the time origin, exclusion of patients with Child–Pugh class B, exclusion of early local therapy, AFP-response thresholds of 30% and 50%, and deterioration in standard ALBI grade. Additional analyses evaluated continuous ALBI change, interaction between the non-favorable phenotype and the two major systemic regimen classes, interaction with baseline modified ALBI stratum, and cause-specific mortality with competing deaths censored at the time of death. Subgroup analyses used the combined non-favorable exposure and a multiplicative interaction term for each subgroup variable. Proportional hazards assumptions were examined using Schoenfeld residuals. No formal multiplicity adjustment was applied because secondary, subgroup, and sensitivity analyses were exploratory; interpretation therefore emphasized effect size, precision, and consistency rather than isolated P values.

## 3 Results

### 3.1 Cohort construction and phenotype distribution

The source cohort comprised 286 patients. Sixty-eight of 286 patients (23.8%) had baseline AFP below 20 ng/mL and were not eligible for the AFP-response phenotype. Among the remaining 218 patients, 185 had complete AFP, albumin, and bilirubin data during days 28–70 for phenotype assignment. Nineteen were not event-free or under follow-up at day 70, leaving 166 patients in the primary landmark cohort (**Figure 1**; **Supplementary Table S2**). The joint phenotype-imaging cohort included 165 patients. Within this cohort, 79 patients had RECIST stable disease and 59 had mRECIST stable disease (**Supplementary Table S11**). A descriptive comparison of the 166 included patients with the 120 excluded or non-analytic patients is provided in **Supplementary Table S3**; crude outcomes in that table are reported from treatment initiation and are not estimates of the day-70 landmark estimand.

**Figure 1:**
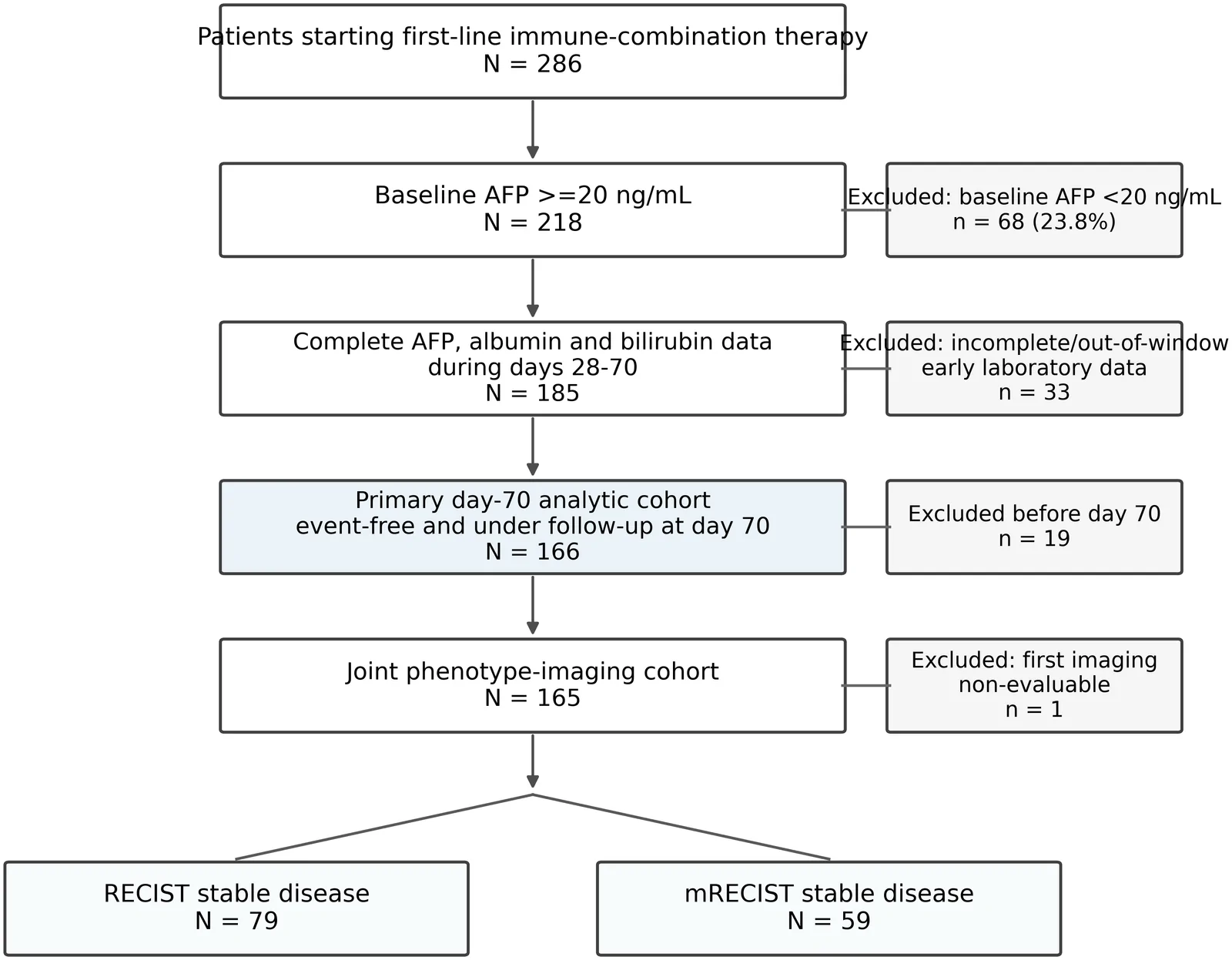
Study design and landmark framework. The flow diagram shows construction of the source cohort, including the 68 of 286 patients (23.8%) with baseline AFP below 20 ng/mL, the phenotype-evaluable cohort with laboratory measurements obtained during days 28–70, the primary day-70 analytic cohort, and the phenotype-imaging cohort with evaluable first imaging. The RECIST and mRECIST stable-disease groups were nested exploratory subgroups. Follow-up for the primary survival analysis began on day 70; survival analyses within imaging-defined stable disease began on the later of day 70 or the first imaging date.

In the primary cohort, 58 patients (34.9%) were classified as concordant favorable, 36 (21.7%) as response-liver vulnerability, 43 (25.9%) as nonresponse-liver preserved, and 29 (17.5%) as concordant failure (**Table 1**; **Figure 2**).

**Table 1:** Early AFP-ALBI response-tolerance assessment and phenotype components.

Characteristic	Overall (n = 166)	Concordant favorable (n = 58)	Response-liver vulnerability (n = 36)	Nonresponse-liver preserved (n = 43)	Concordant failure (n = 29)	P-value
Timing and AFP dynamics						
Landmark laboratory day from treatment start	44.0 (39.0–50.0)	43.0 (37.2–49.0)	45.5 (39.0–50.0)	45.0 (39.0–50.5)	46.0 (39.0–50.0)	0.905
Landmark AFP, ng/mL	483.2 (161.6–2,459.3)	368.2 (108.0–1,952.1)	308.1 (104.0–665.2)	1,406.5 (483.8–4,194.6)	1,182.8 (266.9–4,652.4)	<0.001
AFP change from baseline, %	−27.6 (−53.3 to −5.3)	−52.1 (−64.8 to −40.7)	−50.3 (−60.3 to −39.0)	26.2 (−7.4 to 109.1)	−9.1 (−19.0 to 48.5)	<0.001
AFP response (at least 20% decline)	94/166 (56.6%)	58/58 (100.0%)	36/36 (100.0%)	0/43 (0.0%)	0/29 (0.0%)	By definition
Modified ALBI grade at baseline						
Grade 1	29/166 (17.5%)	14/58 (24.1%)	3/36 (8.3%)	9/43 (20.9%)	3/29 (10.3%)	
Grade 2a	46/166 (27.7%)	21/58 (36.2%)	8/36 (22.2%)	12/43 (27.9%)	5/29 (17.2%)	
Grade 2b	89/166 (53.6%)	22/58 (37.9%)	25/36 (69.4%)	21/43 (48.8%)	21/29 (72.4%)	
Grade 3	2/166 (1.2%)	1/58 (1.7%)	0/36 (0.0%)	1/43 (2.3%)	0/29 (0.0%)	
Modified ALBI grade at landmark						
Grade 1	31/166 (18.7%)	18/58 (31.0%)	0/36 (0.0%)	13/43 (30.2%)	0/29 (0.0%)	
Grade 2a	38/166 (22.9%)	21/58 (36.2%)	3/36 (8.3%)	11/43 (25.6%)	3/29 (10.3%)	
Grade 2b	49/166 (29.5%)	18/58 (31.0%)	8/36 (22.2%)	18/43 (41.9%)	5/29 (17.2%)	
Grade 3	48/166 (28.9%)	1/58 (1.7%)	25/36 (69.4%)	1/43 (2.3%)	21/29 (72.4%)	
ALBI score change	0.09 (−0.05 to 0.52)	−0.03 (−0.13 to 0.07)	0.58 (0.37 to 0.80)	−0.03 (−0.09 to 0.01)	0.68 (0.51 to 0.88)	<0.001
Modified ALBI deterioration	65/166 (39.2%)	0/58 (0.0%)	36/36 (100.0%)	0/43 (0.0%)	29/29 (100.0%)	By definition

Values are median (IQR) or n/N (%) unless otherwise stated. AFP response and modified ALBI deterioration define the phenotypes; inferential comparisons for these rows are therefore not interpreted. Modified ALBI deterioration was defined as worsening by at least one modified ALBI category from baseline to the early assessment.

**Figure 2:**
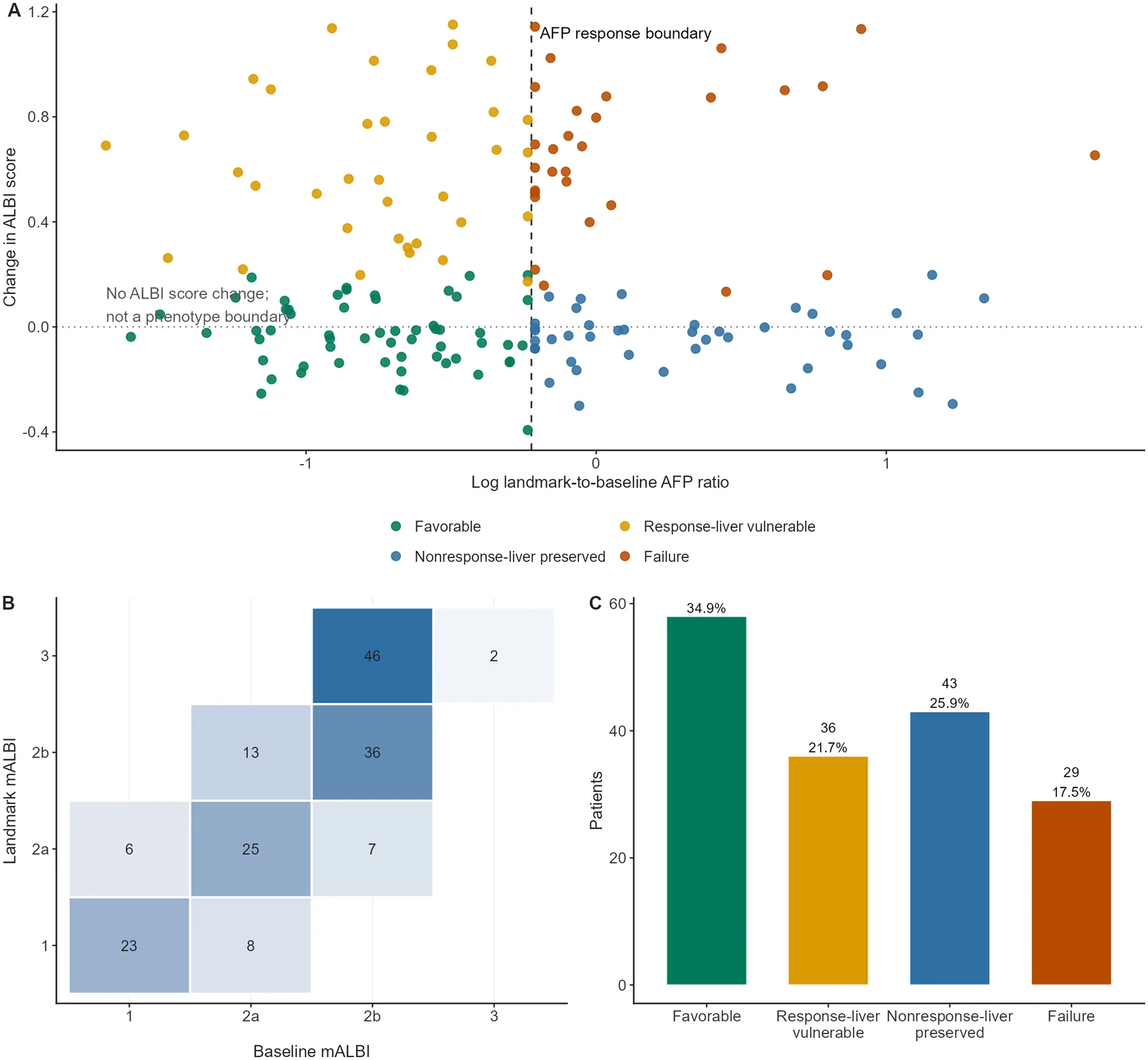
Construction of AFP-ALBI response-tolerance phenotypes. **(A)** Scatter plot of the log landmark-to-baseline AFP ratio against change in ALBI score. The vertical dashed line marks an AFP ratio of 0.80. The horizontal line marks no change in ALBI score and was not used to define phenotype status. **(B)** Transition matrix from baseline to landmark modified ALBI grade. **(C)** Distribution of the four phenotypes. Shortened labels are used in the figure for readability.

### 3.2 Baseline characteristics

The median age was 58.4 years, and 144 of 166 patients (86.7%) were men. Ninety-five patients (57.2%) had BCLC stage C disease, 87 (52.4%) had macrovascular invasion, and 60 (36.1%) had extrahepatic metastases (**Table 2**). Baseline characteristics varied across phenotypes. Macrovascular invasion was most common in concordant failure (79.3%), and tumor burden scores were higher in the two AFP-nonresponse groups. Child–Pugh class B was present in 72.2% of patients with response-liver vulnerability and 75.9% of those with concordant failure, compared with 44.8% of concordant favorable and 30.2% of nonresponse-liver preserved patients. Baseline ALBI scores also differed across phenotypes. These imbalances motivated direct adjustment for baseline liver reserve and measured tumor burden in the primary model and limit causal interpretation of the phenotype-outcome associations.

**Table 2:** Baseline characteristics of the day-70 AFP-evaluable analytic cohort by AFP-ALBI phenotype.

Characteristic	Overall (n = 166)	Concordant favorable (n = 58)	Response-liver vulnerability (n = 36)	Nonresponse-liver preserved (n = 43)	Concordant failure (n = 29)	P-value
Demographics and clinical status						
Age, years	58.4 (52.4–64.5)	58.7 (51.2–63.8)	58.3 (52.7–66.0)	60.9 (55.0–65.8)	56.8 (52.5–62.9)	0.437
Male sex	144/166 (86.7%)	49/58 (84.5%)	32/36 (88.9%)	37/43 (86.0%)	26/29 (89.7%)	0.935
ECOG performance status						0.175
0	41/166 (24.7%)	13/58 (22.4%)	11/36 (30.6%)	14/43 (32.6%)	3/29 (10.3%)	
1	107/166 (64.5%)	35/58 (60.3%)	22/36 (61.1%)	26/43 (60.5%)	24/29 (82.8%)	
2	18/166 (10.8%)	10/58 (17.2%)	3/36 (8.3%)	3/43 (7.0%)	2/29 (6.9%)	
Tumor stage and burden						
BCLC stage						0.087
B	71/166 (42.8%)	28/58 (48.3%)	19/36 (52.8%)	17/43 (39.5%)	7/29 (24.1%)	
C	95/166 (57.2%)	30/58 (51.7%)	17/36 (47.2%)	26/43 (60.5%)	22/29 (75.9%)	
Macrovascular invasion	87/166 (52.4%)	25/58 (43.1%)	21/36 (58.3%)	18/43 (41.9%)	23/29 (79.3%)	0.004
Extrahepatic metastasis	60/166 (36.1%)	19/58 (32.8%)	12/36 (33.3%)	15/43 (34.9%)	14/29 (48.3%)	0.537
Tumor burden score	10.3 (7.9–13.3)	9.8 (7.6–12.6)	9.2 (6.6–10.9)	11.7 (8.9–14.6)	11.3 (9.1–4.8)	0.033
Liver reserve and etiology						
Child–Pugh class						<0.001
A	79/166 (47.6%)	32/58 (55.2%)	10/36 (27.8%)	30/43 (69.8%)	7/29 (24.1%)	
B	87/166 (52.4%)	26/58 (44.8%)	26/36 (72.2%)	13/43 (30.2%)	22/29 (75.9%)	
HBV-related etiology	130/166 (78.3%)	44/58 (75.9%)	29/36 (80.6%)	33/43 (76.7%)	24/29 (82.8%)	0.900
Baseline AFP, ng/mL	747.6 (263.2–2,611.8)	747.6 (263.2–3,107.5)	496.2 (242.1–1,770.2)	834.2 (358.6–2,654.5)	971.0 (329.5–3,217.8)	0.485
Baseline ALBI score	−2.22 (−2.51 to −1.98)	−2.40 (−2.58 to −2.18)	−2.04 (−2.36 to −1.70)	−2.23 (−2.52 to −2.03)	−2.02 (−2.29 to −1.84)	<0.001
Treatment context						
Systemic regimen class						0.841
Dual ICI/other ICI combination	12/166 (7.2%)	4/58 (6.9%)	2/36 (5.6%)	5/43 (11.6%)	1/29 (3.4%)	
ICI + anti-VEGF antibody	69/166 (41.6%)	25/58 (43.1%)	13/36 (36.1%)	19/43 (44.2%)	12/29 (41.4%)	
ICI + TKI	85/166 (51.2%)	29/58 (50.0%)	21/36 (58.3%)	19/43 (44.2%)	16/29 (55.2%)	
Early local therapy within 8 weeks	42/166 (25.3%)	12/58 (20.7%)	11/36 (30.6%)	11/43 (25.6%)	8/29 (27.6%)	0.728

Values are median (IQR) or n/N (%) unless otherwise stated. P-values are descriptive and should not be interpreted as evidence of balance between non-randomized groups. AFP, alpha-fetoprotein; ALBI, albumin-bilirubin; BCLC, Barcelona Clinic Liver Cancer; ECOG, Eastern Cooperative Oncology Group; HBV, hepatitis B virus; ICI, immune checkpoint inhibitor; TKI, tyrosine kinase inhibitor; VEGF, vascular endothelial growth factor.

### 3.3 Early AFP and liver-reserve assessment

The early laboratory assessment was obtained a median of 44.0 days after treatment initiation (IQR, 39.0–50.0) (**Table 1**). Ninety-four patients (56.6%) met the AFP-response criterion, and 65 (39.2%) experienced deterioration in modified ALBI grade. At baseline, modified ALBI grade 2b was present in 25 of 36 patients (69.4%) with response-liver vulnerability and 21 of 29 (72.4%) with concordant failure, compared with 22 of 58 (37.9%) with concordant favorable. At the landmark assessment, modified ALBI grade 3 was present in 25 of 36 (69.4%) and 21 of 29 (72.4%) patients in the two deterioration phenotypes, respectively (**Table 1**; **Figure 2**). Because categorical worsening can depend on the starting grade and proximity to a category boundary, baseline continuous ALBI was included in the primary model and continuous ALBI change was examined in sensitivity analysis.

### 3.4 Imaging response, post-landmark outcomes, and safety

The first post-treatment scan was performed a median of 66.0 days after treatment initiation. In the joint phenotype-imaging cohort, RECIST 1.1 classified 2 patients (1.2%) as complete response, 45 (27.3%) as partial response, 79 (47.9%) as stable disease, and 39 (23.6%) as progressive disease (**Table 3**; **Figure 3**). The corresponding mRECIST counts were 4 (2.4%), 67 (40.6%), 59 (35.8%), and 35 (21.2%). Progressive disease was most frequent in concordant failure by both RECIST 1.1 (44.8%) and mRECIST (41.4%).

**Table 3:** First imaging response among patients with evaluable post-treatment imaging.

Characteristic	Overall (n = 165)	Concordant favorable (n = 57)	Response-liver vulnerability (n = 36)	Nonresponse-liver preserved (n = 43)	Concordant failure (n = 29)	P-value
First imaging timing	66.0 (59.0–75.0)	66.0 (58.0–75.0)	68.5 (57.5–76.5)	63.0 (59.0–71.0)	69.0 (61.0–81.0)	0.331
RECIST 1.1 response						
Complete response	2/165 (1.2%)	2/57 (3.5%)	0/36 (0.0%)	0/43 (0.0%)	0/29 (0.0%)	
Partial response	45/165 (27.3%)	16/57 (28.1%)	13/36 (36.1%)	12/43 (27.9%)	4/29 (13.8%)	
Stable disease	79/165 (47.9%)	30/57 (52.6%)	18/36 (50.0%)	19/43 (44.2%)	12/29 (41.4%)	
Progressive disease	39/165 (23.6%)	9/57 (15.8%)	5/36 (13.9%)	12/43 (27.9%)	13/29 (44.8%)	
mRECIST response						
Complete response	4/165 (2.4%)	2/57 (3.5%)	0/36 (0.0%)	2/43 (4.7%)	0/29 (0.0%)	
Partial response	67/165 (40.6%)	24/57 (42.1%)	22/36 (61.1%)	14/43 (32.6%)	7/29 (24.1%)	
Stable disease	59/165 (35.8%)	23/57 (40.4%)	10/36 (27.8%)	16/43 (37.2%)	10/29 (34.5%)	
Progressive disease	35/165 (21.2%)	8/57 (14.0%)	4/36 (11.1%)	11/43 (25.6%)	12/29 (41.4%)	

First imaging response was summarized among primary-cohort patients with evaluable imaging. For survival analyses restricted to imaging-defined stable disease, the joint time origin was the later of day 70 or the first imaging date. Values are median (IQR) or n/N (%). CR, complete response; mRECIST, modified Response Evaluation Criteria in Solid Tumors; PD, progressive disease; PR, partial response; RECIST, Response Evaluation Criteria in Solid Tumors; SD, stable disease.

**Figure 3:**
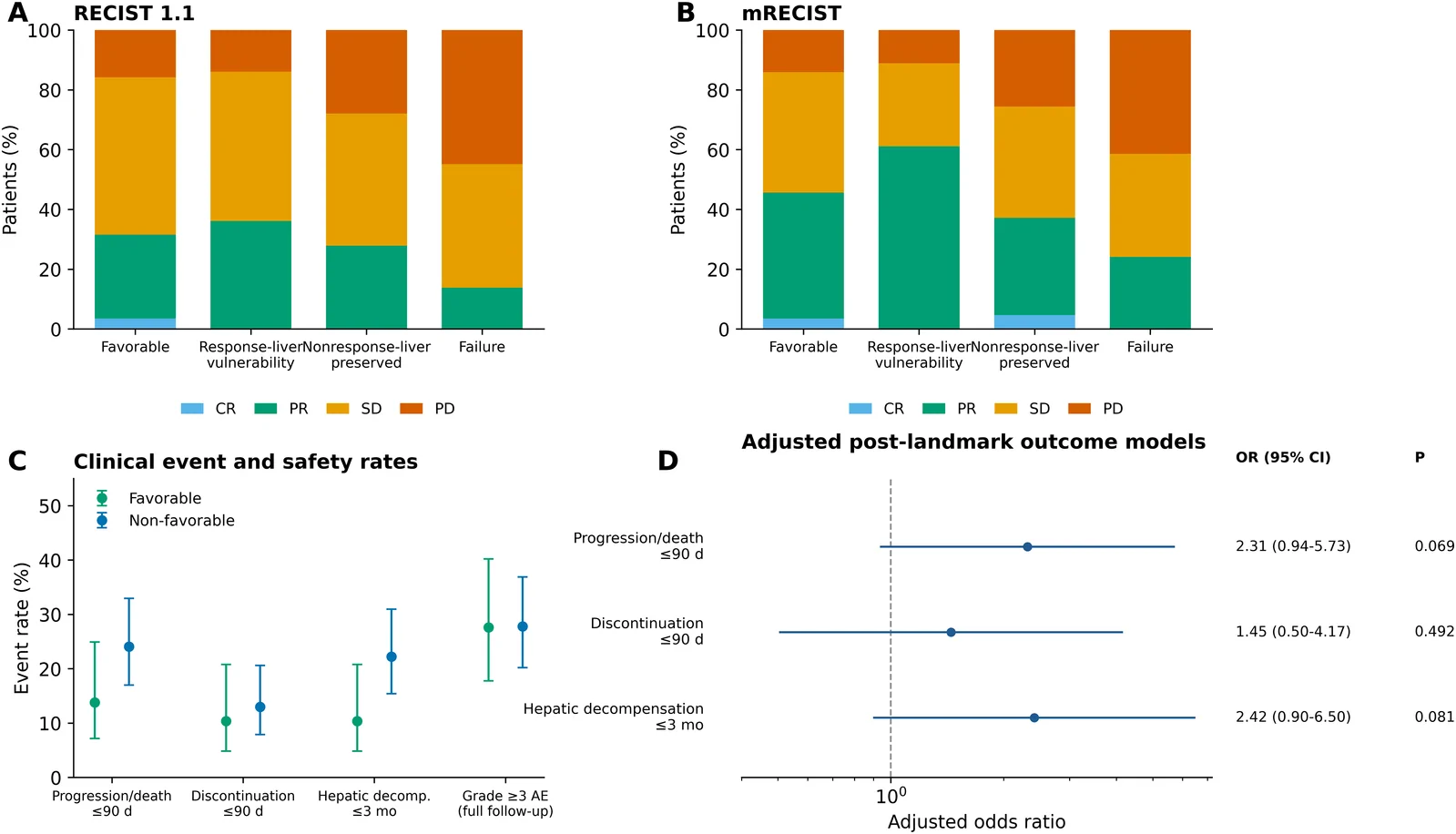
Imaging response, post-landmark outcomes, and treatment-period safety. **(A)** RECIST 1.1 response distribution by phenotype. **(B)** mRECIST response distribution by phenotype. **(C)** Rates with 95% confidence intervals for the three post-landmark clinical outcomes and grade 3 or higher adverse events recorded over the entire treatment follow-up. **(D)** Adjusted odds ratios for the three post-landmark secondary outcomes comparing the non-favorable group with concordant favorable, adjusted for baseline continuous ALBI score. Grade 3 or higher adverse events are shown descriptively in **(C)** and are not included in **(D)** because their recording window spans the entire treatment follow-up rather than beginning at day 70. CI, confidence interval; OR, odds ratio.

In parsimonious adjusted post-landmark analyses, the estimate for hepatic decompensation within 3 months after day 70 was elevated but imprecise (odds ratio (OR) 2.42, 95% CI 0.90–6.50; P = 0.081). Estimates for progression or death within 90 days (OR 2.31, 95% CI 0.94–5.73; P = 0.069) and treatment discontinuation within 90 days (OR 1.45, 95% CI 0.50–4.17; P = 0.492) were also imprecise and included the null value (**Table 4**; **Figure 3**). These models compare the combined non-favorable group with concordant favorable and do not identify risk attributable to any single discordant phenotype. Across the entire treatment follow-up, grade 3 or higher adverse events were recorded in 46 of 166 patients (27.7%); descriptive rates were 16 of 58 (27.6%) in concordant favorable and 30 of 108 (27.8%) in the combined non-favorable group (**Figure 3**).

**Table 4:** Secondary post-landmark clinical outcome models.

Outcome	N	Events	Adjusted OR (95% CI)	P-value
Progression or death within 90 days after day-70 landmark	166	34	2.31 (0.94–5.73)	0.069
Treatment discontinuation within 90 days after day-70 landmark	166	20	1.45 (0.50–4.17)	0.492
Hepatic decompensation within 3 months after day-70 landmark	166	30	2.42 (0.90–6.50)	0.081

Effect estimates compare the non-favorable AFP-ALBI group with concordant favorable. Each model included the combined phenotype exposure and baseline continuous ALBI score. CI, confidence interval; OR, odds ratio.

### 3.5 Landmark survival outcomes

After the day-70 landmark, 130 PFS events and 64 deaths were recorded. In unadjusted analyses, PFS was shorter for nonresponse-liver preserved (HR 1.90, 95% CI 1.19–3.03; P = 0.007) and concordant failure (HR 3.41, 95% CI 2.02–5.74; P <0.001) than for concordant favorable. The estimate for response-liver vulnerability was 1.60 (95% CI 0.99–2.59; P = 0.057) (**Table 5**; **Figure 4**).

**Table 5:** Landmark Cox models for progression-free survival and overall survival.

Outcome	Model	Phenotype contrast	N	Events	HR (95% CI)	P-value
Progression-free survival	Unadjusted four-class	Response-liver vulnerability vs. concordant favorable	166	130	1.60 (0.99–2.59)	0.057
		Nonresponse-liver preserved vs. concordant favorable	166	130	1.90 (1.19–3.03)	0.007
		Concordant failure vs. concordant favorable	166	130	3.41 (2.02–.74)	<0.001
	Adjusted four-class	Response-liver vulnerability vs. concordant favorable	166	130	1.49 (0.89–.49)	0.130
		Nonresponse-liver preserved vs. concordant favorable	166	130	1.69 (1.02–2.79)	0.040
		Concordant failure vs. concordant favorable	166	130	2.85 (1.63–4.99)	<0.001
	Adjusted non-favorable	Non-favorable vs. concordant favorable	166	130	1.79 (1.18–2.72)	0.006
Overall survival	Unadjusted four-class	Response-liver vulnerability vs. concordant favorable	166	64	1.38 (0.69–2.74)	0.363
		Nonresponse-liver preserved vs. concordant favorable	166	64	1.01 (0.50–2.06)	0.970
		Concordant failure vs. concordant favorable	166	64	2.52 (1.28–.96)	0.008
	Simplified adjusted four-class	Response-liver vulnerability vs. concordant favorable	166	64	1.13 (0.53–2.43)	0.753
		Nonresponse-liver preserved vs. concordant favorable	166	64	0.95 (0.46–1.95)	0.888
		Concordant failure vs. concordant favorable	166	64	2.09 (1.00–4.38)	0.050
	Simplified adjusted non-favorable	Non-favorable vs. concordant favorable	166	64	1.23 (0.68–2.22)	0.503

Hazard ratios compare each phenotype with concordant favorable unless otherwise stated. The adjusted PFS model included phenotype, ECOG performance status, macrovascular invasion, extrahepatic metastasis, tumor burden score, baseline continuous ALBI score, and systemic regimen class. The reduced OS model included phenotype, ECOG performance status, macrovascular invasion, and baseline continuous ALBI score. CI, confidence interval; HR, hazard ratio; OS, overall survival; PFS, progression-free survival.

**Figure 4:**
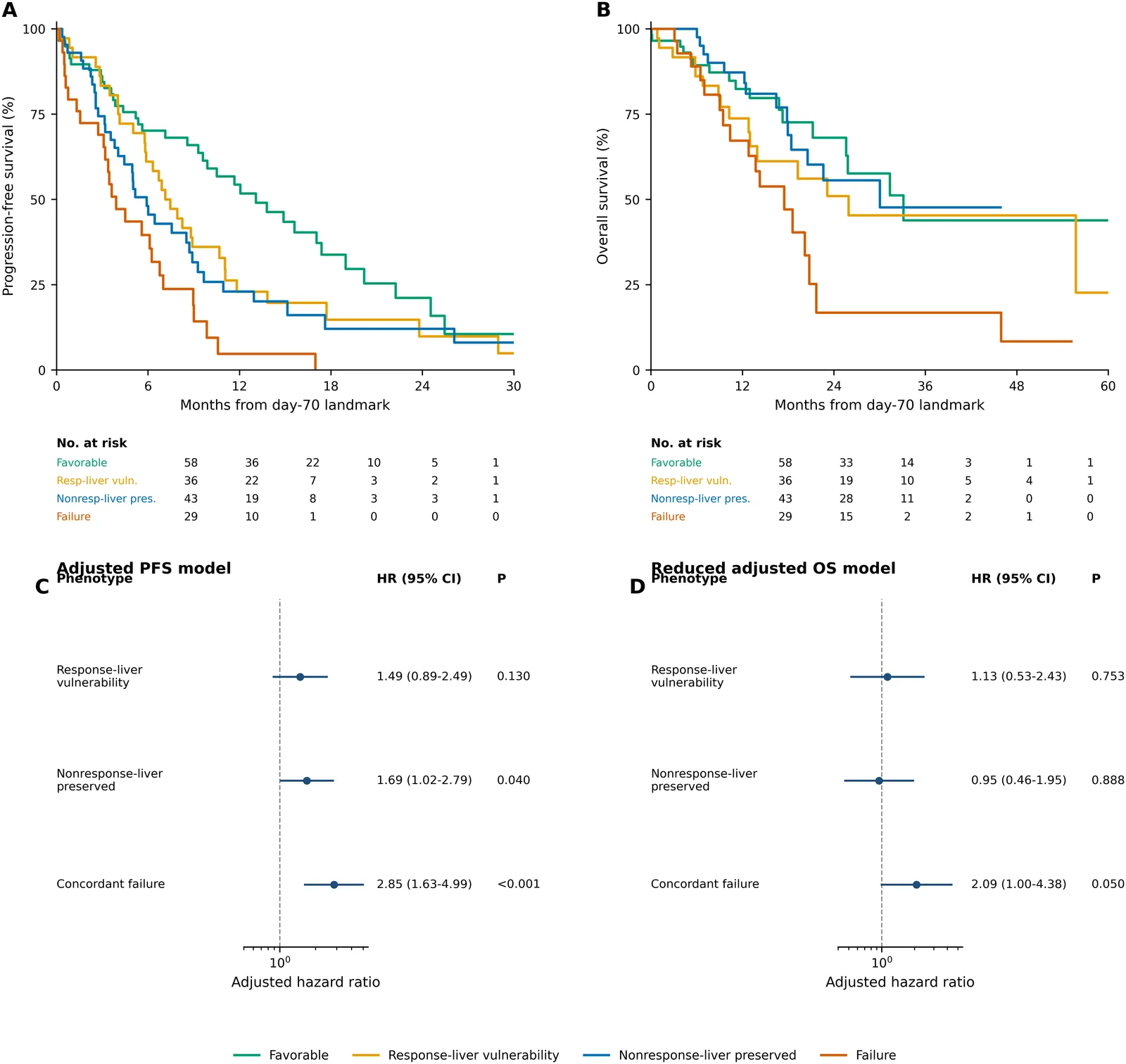
Landmark survival outcomes. Kaplan–Meier curves with numbers at risk show progression-free survival **(A)** and overall survival **(B)** from the day-70 landmark. The adjusted PFS model **(C)** included phenotype, ECOG performance status, macrovascular invasion, extrahepatic metastasis, tumor burden score, baseline continuous ALBI score, and systemic regimen class. The reduced adjusted OS model **(D)** included phenotype, ECOG performance status, macrovascular invasion, and baseline continuous ALBI score. CI, confidence interval; HR, hazard ratio.

After adjustment for ECOG performance status, macrovascular invasion, extrahepatic metastasis, tumor burden score, baseline continuous ALBI score, and systemic regimen class, concordant failure retained the largest association with shorter PFS (HR 2.85, 95% CI 1.63–4.99; P <0.001). Nonresponse-liver preserved also remained associated with shorter PFS (HR 1.69, 95% CI 1.02–2.79; P = 0.040), whereas the estimate for response-liver vulnerability remained imprecise (HR 1.49, 95% CI 0.89–2.49; P = 0.130). In the collapsed analysis, the non-favorable group had an adjusted HR of 1.79 (95% CI 1.18–2.72; P = 0.006) (**Table 5**; **Figure 4**).

OS estimates were less precise. In the reduced adjusted model, concordant failure had the largest point estimate (HR 2.09, 95% CI 1.00–4.38; P = 0.050), but the confidence interval reached the null boundary. Estimates for response-liver vulnerability (HR 1.13, 95% CI 0.53–2.43; P = 0.753), nonresponse-liver preserved (HR 0.95, 95% CI 0.46–1.95; P = 0.888), and the combined non-favorable group (HR 1.23, 95% CI 0.68–2.22; P = 0.503) were imprecise (**Table 5**; **Figure 4**). Accordingly, the OS analysis was treated as exploratory rather than confirmatory.

### 3.6 Sensitivity, subgroup, and stable-disease analyses

The PFS association for the combined non-favorable group was consistent in direction across the day-42 restricted analysis (HR 2.63, 95% CI 1.32–5.21), the actual laboratory-date analysis (HR 1.78, 95% CI 1.19–2.65), exclusion of Child–Pugh class B (HR 1.92, 95% CI 1.03–3.59), exclusion of early local therapy (HR 1.68, 95% CI 1.06–2.65), alternative AFP-response thresholds, and deterioration in standard ALBI grade (**Supplementary Table S7**; **Supplementary Figure S2**). In a continuous analysis, each 0.5-unit increase in ALBI score was associated with shorter PFS after adjustment (HR 1.33, 95% CI 1.04–1.68; P = 0.021), while AFP response remained associated with longer PFS (HR 0.57, 95% CI 0.39–0.83; P = 0.003); the AFP-response by continuous-ALBI-change interaction was not evident (P = 0.828) (**Supplementary Table S12**). OS sensitivity estimates remained imprecise (**Supplementary Table S8**; **Supplementary Figure S4**).

There was no clear evidence that the PFS association differed across the clinical subgroups; interaction P values ranged from 0.236 to 0.966 (**Supplementary Table S9**; **Supplementary Figure S3**). Among the two major systemic regimen classes, the adjusted non-favorable HR was 2.45 (95% CI 1.27–4.72) for ICI plus anti-VEGF antibody and 1.74 (95% CI 1.01–3.00) for ICI plus TKI, with no clear interaction (P = 0.422); the dual-ICI/other-combination group remained too small for stable effect-modification estimates (**Supplementary Tables S10**, **S12**). The association also did not show clear heterogeneity between baseline modified ALBI grades 1/2a and 2b/3 (P for interaction = 0.814). Cause-specific mortality analyses were limited by 40 HCC-progression deaths and 15 liver-failure deaths and yielded wide confidence intervals (**Supplementary Table S12**). Among patients with RECIST stable disease, phenotype-specific PFS estimates remained exploratory (**Figure 5**; **Supplementary Table S11**; **Supplementary Figure S5**). Missingness and RECIST inter-reader agreement are summarized in **Supplementary Tables S4**, **S5**; **Supplementary Figure S1**. Proportional-hazards diagnostics are shown in **Supplementary Figure S6**.

**Figure 5:**
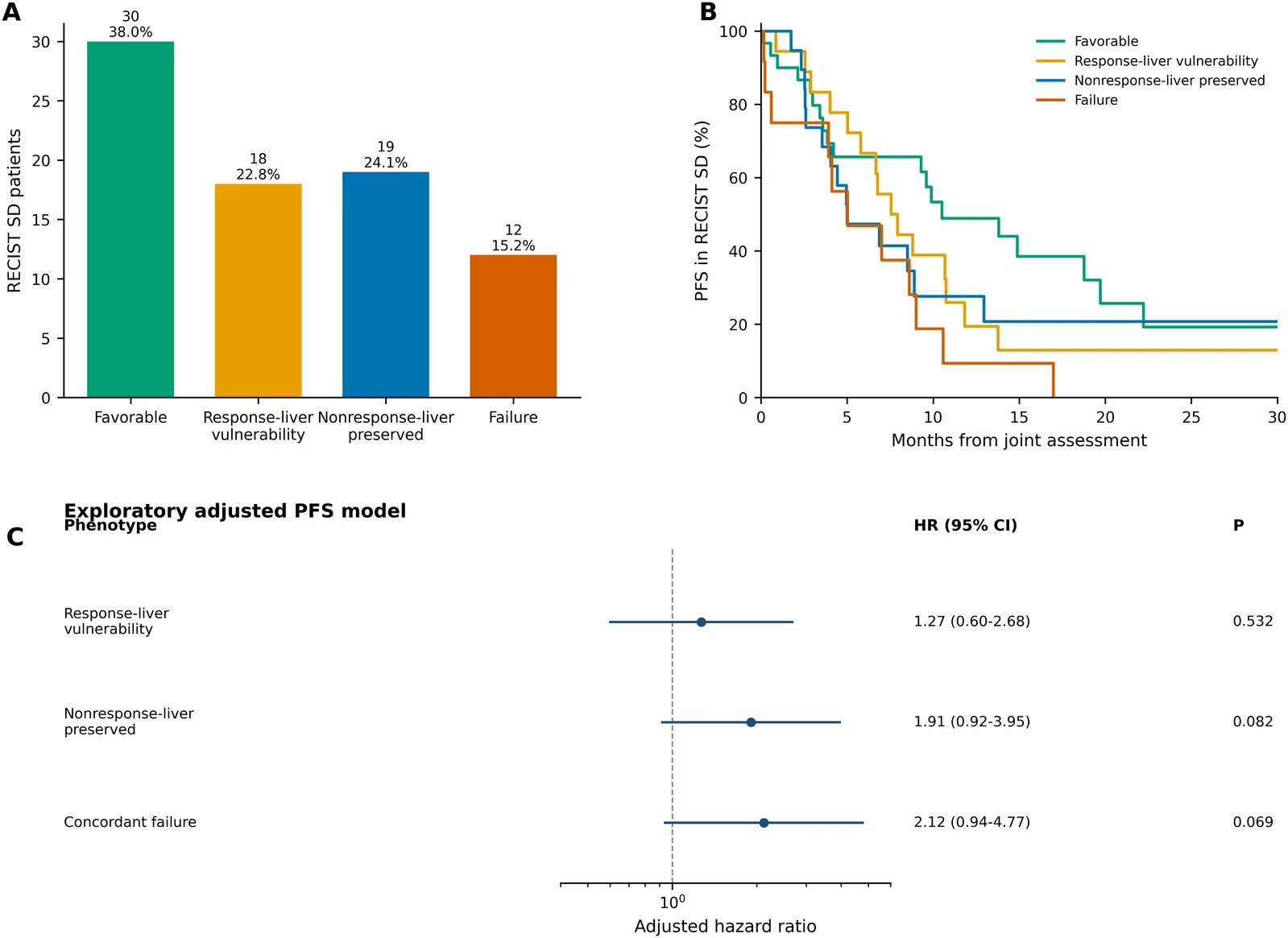
Exploratory analysis among patients with RECIST stable disease. The figure presents phenotype distribution **(A)**, progression-free survival from the joint phenotype-imaging landmark **(B)**, and exploratory adjusted hazard-ratio estimates **(C)** among patients whose first scan showed RECIST stable disease. The adjusted model included phenotype, ECOG performance status, macrovascular invasion, and baseline continuous ALBI score. These analyses were hypothesis-generating and were not interpreted as treatment-decision rules. CI, confidence interval; HR, hazard ratio; SD, stable disease.

## 4 Discussion

This analysis treated early AFP kinetics and modified ALBI change as a paired assessment of tumor-marker response and hepatic tolerance. Concordant failure, defined by absent AFP response and deterioration in modified ALBI grade, showed the largest and most consistent association with subsequent PFS. Nonresponse-liver preserved was also associated with shorter PFS, indicating that maintained hepatic reserve should not be equated with effective tumor control. By contrast, response-liver vulnerability did not show a clear independent PFS association after adjustment. OS contained only 64 deaths; although the largest point estimate again occurred in concordant failure, the interval reached the null boundary and the OS findings remain inconclusive. The discordant phenotypes therefore appear to describe different clinical states, but their prognostic separation is clearest for PFS rather than OS.

This distinction is particularly relevant in HCC, where systemic treatment is delivered against a background of cirrhosis, portal hypertension, chronic inflammation, or viral hepatitis, and clinical deterioration may arise from both tumor progression and liver failure (3, 5, 33). ALBI was developed to quantify hepatic function without the subjective elements of the Child–Pugh score and has since shown prognostic value across HCC treatment settings (24–26). Modified ALBI further separates the intermediate range, in which relatively small biochemical changes may affect treatment feasibility (27, 28). Our findings extend this approach from baseline staging to early on-treatment monitoring by asking whether tumor-marker response and hepatic reserve improve, worsen, or diverge over the same treatment interval.

Concordant failure is a clinically plausible high-risk pattern. In AFP-evaluable HCC, failure of AFP to decline is compatible with ongoing tumor activity, while worsening modified ALBI grade signals a narrowing hepatic therapeutic window. Either process can shorten PFS. Patients with concordant failure also had more macrovascular invasion and poorer liver reserve at baseline, and the phenotype therefore cannot be interpreted as a treatment-emergent causal state. Adjustment for baseline continuous ALBI, measured tumor burden, and clinical status reduces but cannot eliminate this concern. The observed association may still partly reflect baseline disease severity that is carried forward into the early on-treatment phenotype.

Nonresponse-liver preserved carries a different implication. These patients maintained hepatic reserve but did not reach the AFP-response threshold. Preserved liver function may allow treatment to continue, particularly when the first scan shows stable disease, but it does not demonstrate biological tumor control. Previous studies have linked early AFP decline with imaging response and survival during ICI-based and antiangiogenic therapy (17–22), and pooled evidence supports a 20% early decline as a pragmatic threshold (23). The persistence of the PFS association in this group is consistent with AFP kinetics capturing an oncologic signal that tolerability alone does not provide. Clinically, this pattern supports timely reassessment rather than automatic treatment discontinuation.

Response-liver vulnerability was more difficult to interpret. Because AFP declined, this group should not be described as uniform treatment failure, and its adjusted PFS estimate remained compatible with little or no independent association. The clinically relevant feature was the divergence between apparent tumor-marker response and worsening hepatic reserve. The hepatic-decompensation model combined all non-favorable phenotypes and produced an imprecise estimate after parsimonious adjustment; it therefore cannot attribute decompensation risk specifically to this discordant group. The pattern nevertheless cautions against viewing an AFP decline as wholly reassuring when liver function is worsening. Treatment decisions still require assessment of the cause and reversibility of hepatic deterioration, treatment exposure, portal-hypertension complications, local therapy, and imaging.

Most prior studies have examined AFP and ALBI separately. AFP response has been evaluated in ICI-based cohorts and atezolizumab-bevacizumab series, although thresholds and sampling times have varied (17–23). ALBI and modified ALBI have been used mainly for baseline prognostic assessment and treatment eligibility (24–28). Campani and colleagues combined baseline ALBI with early AFP variation in patients receiving atezolizumab-bevacizumab, providing an important precedent for integrated assessment (21). Compared with approaches that pair early AFP change with baseline liver reserve, the present framework evaluates whether tumor-marker response and hepatic-reserve trajectory evolve concordantly over the same early treatment interval, with outcome follow-up beginning only after that joint state is established. This distinction is most relevant when AFP and liver function move in different directions, because it separates insufficient tumor control from declining hepatic reserve rather than combining the two signals into a baseline-plus-response index.

The findings also need to be viewed within a heterogeneous treatment landscape. Immune-combination regimens differ in therapeutic target, antiangiogenic intensity, toxicity, and regional availability (7–13). Negative phase 3 results for some ICI-TKI combinations show that efficacy cannot be assumed across regimens that appear superficially similar (34), while lenvatinib remains a widely used antiangiogenic backbone in real-world combinations (35). We adjusted for systemic regimen class, but the study was not designed to compare treatments. The dual-ICI/other-combination subgroup was too small for reliable effect-modification analysis, and these data do not support favoring one regimen over another. In the exploratory comparison restricted to ICI plus TKI and ICI plus anti-VEGF antibody, the non-favorable association was directionally similar and the interaction was not evident (P = 0.422), but the confidence intervals were wide enough that clinically relevant heterogeneity cannot be excluded.

Imaging remains indispensable. RECIST 1.1 and mRECIST capture different aspects of treatment response, and neither should be replaced by a serum marker (14–16). We examined stable disease because this category often creates the greatest uncertainty at the first assessment. The joint landmark ensured that both imaging and AFP-ALBI phenotype were known before follow-up began. Even with this safeguard, estimates in the stable-disease subgroup were imprecise and should be regarded as hypothesis-generating. The most useful role for AFP-ALBI status in this setting may be to inform how soon a patient should undergo multimodal reassessment. When the first scan is stable or otherwise equivocal, a non-favorable biochemical pattern may support an earlier review of imaging, liver-function trajectory, toxicity, and treatment feasibility, but not automatic discontinuation or switching.

The landmark design addresses an important source of bias by preventing follow-up from beginning before a post-baseline phenotype can be assigned. Guarantee-time and immortal-time bias can materially distort associations when response groups are defined using later information (29–31). Similar PFS estimates in the fixed day-70, day-42 restricted, and actual laboratory-date analyses make it less likely that the principal finding depended on a single arbitrary time origin. Additional strengths include direct recalculation of AFP and ALBI components, separation of systemic regimen class from early local therapy, parallel RECIST and mRECIST descriptions, and sensitivity analyses using alternative phenotype definitions. The direction of the finding was also retained when ALBI was analyzed continuously, which makes it less likely that the PFS association was created solely by modified-ALBI category boundaries.

The study also has important limitations. Its retrospective, single-center design leaves substantial scope for residual confounding, and the phenotype may partly encode baseline liver reserve and tumor burden despite direct adjustment for continuous baseline ALBI and measured tumor features. The primary estimand was restricted to AFP-evaluable patients who remained event-free through day 70: 68 of 286 treated patients (23.8%) had baseline AFP below 20 ng/mL, and a further 52 did not enter the day-70 analytic cohort because of incomplete early laboratory assessment or failure to remain event-free and under follow-up through day 70. The framework therefore does not describe AFP-nonproducing HCC or the earliest treatment failures. Categorical modified-ALBI deterioration is clinically interpretable but can be sensitive to category boundaries and starting grade. Although the continuous-change and baseline-grade interaction analyses were concordant with the main PFS result, residual dependence on baseline liver reserve remains possible. Treatment regimens were heterogeneous, and the interaction comparison between the two major regimen classes remained exploratory. OS was limited by 64 deaths, and cause-specific analyses were particularly sparse, including only 15 liver-failure deaths. No formal multiplicity adjustment was applied to the secondary, subgroup, and sensitivity analyses. Finally, the stable-disease analyses were exploratory and lacked external validation.

Prospective studies should standardize the timing of AFP, albumin, bilirubin, treatment-exposure, decompensation, and imaging assessments. Incorporating des-gamma-carboxy prothrombin (DCP)/protein induced by vitamin K absence or antagonist-II (PIVKA-II) may extend the approach to patients with low AFP. External cohorts with different dominant first-line regimens are needed to test transportability, while longitudinal models could determine whether recovery from, or persistence of, liver deterioration adds prognostic information. A useful monitoring strategy should ultimately answer three separate questions: whether the tumor is responding, whether the liver can tolerate continued therapy, and whether the patient remains eligible for subsequent treatment.

## 5 Conclusion

Among AFP-evaluable patients with unresectable HCC who remained event-free through the day-70 landmark, early discordance between AFP response and modified ALBI deterioration distinguished clinically different response-tolerance patterns. Concordant failure had the strongest association with shorter PFS, and AFP nonresponse despite preserved hepatic reserve was also associated with shorter PFS. The main practical implication is closer and earlier multimodal reassessment when biochemical findings remain non-favorable despite an equivocal first imaging assessment, rather than phenotype-based treatment switching. External prospective validation is required before clinical implementation.

## Data Availability

The original contributions presented in the study are included in the article/**Supplementary Material**. Further inquiries can be directed to the corresponding author.
