# Supplementary material for: Early discordance between alpha-fetoprotein response and albumin-bilirubin deterioration refines response assessment in unresectable hepatocellular carcinoma receiving first-line immune-combination therapy: a real-world landmark cohort study
Source: Front Oncol. 2026 Oct 19;16:1954746. doi: 10.3389/fonc.2026.1954746 (PMC13724454; doi:10.3389/fonc.2026.1954746)
Supplement: Supplementary file 1 [file SupplementaryFile1.docx]

# Supplementary Methods

The primary analysis used a fixed day-70 landmark. Patients with baseline AFP <20 ng/mL were considered structurally non-applicable for AFP-response phenotyping rather than having a missing phenotype. The early laboratory window spanned days 28-70 after treatment initiation. Exposure-defining AFP, albumin, and bilirubin values were not imputed.

The day-42 restricted sensitivity analysis used only complete laboratory values obtained from day 28 through day 42 and began follow-up on day 42. The actual laboratory-date sensitivity analysis used the recorded landmark laboratory date as the pragmatic phenotype-assessment date because separate dates for AFP, albumin, and bilirubin were unavailable in the analysis file. Imaging-adjusted and stable-disease analyses used the later of day 70 and the first imaging date as the joint time origin.

The primary phenotype used an AFP decline of at least 20% and deterioration of at least one modified ALBI category. Sensitivity analyses used AFP declines of at least 30% and 50% and standard ALBI-grade deterioration. The collapsed non-favorable phenotype included response-liver vulnerability, nonresponse-liver preserved, and concordant failure.

Subgroup analyses were exploratory. Each model included a multiplicative interaction term between the collapsed phenotype and one subgroup variable. P for interaction was displayed once for each subgroup category. Regimen-specific interpretation was limited because the dual-ICI/other-combination stratum was sparse.

**Supplementary Table S1. Analysis definitions and derived variables**

| **Concept** | **Definition** |
| --- | --- |
| Primary day-70 landmark | Treatment start date plus 70 days; primary survival time origin. |
| Day-42 restricted sensitivity landmark | Treatment start date plus 42 days; uses only day 28-42 laboratory values. |
| Actual laboratory-date sensitivity landmark | Landmark laboratory date; used as a pragmatic phenotype-assessment date because separate AFP, albumin, and bilirubin dates were not available. |
| Joint phenotype-imaging landmark | Maximum of the primary day-70 landmark and first imaging date; used as the time origin for survival analyses restricted to imaging-defined stable disease. |
| AFP response | Landmark AFP / baseline AFP <=0.80 among patients with baseline AFP >=20 ng/mL. |
| Modified ALBI deterioration | Any worsening in modified ALBI grade from baseline to the landmark laboratory assessment. |
| AFP-ALBI phenotype | Four-category response-tolerance phenotype defined jointly by AFP response and modified ALBI deterioration. |
| Non-favorable phenotype | All AFP-ALBI phenotypes other than concordant favorable. |
| Systemic regimen class | Systemic therapy reclassified as ICI + TKI, ICI + anti-VEGF antibody, or dual ICI / other ICI combination. |
| Early local therapy within 8 weeks | Binary treatment-process variable retained separately from systemic regimen class. |
| Selection among multiple day 28-70 laboratory assessments | When more than one complete AFP/albumin/total bilirubin assessment was available during days 28-70, the complete assessment closest in calendar time to day 70 was selected. |
| Hepatic decompensation within 3 months after the day-70 landmark | Adjudicated from clinical records on the basis of decompensating events including new or worsening ascites, hepatic encephalopathy, or variceal bleeding. |
| Grade 3 or higher adverse event | Any grade 3 or higher adverse event recorded during the entire treatment follow-up; summarized descriptively and not treated as a post-day-70 outcome. |

AFP, alpha-fetoprotein; ALBI, albumin-bilirubin; ICI, immune-checkpoint inhibitor; TKI, tyrosine kinase inhibitor; VEGF, vascular endothelial growth factor.

**Supplementary Table S2. Cohort construction and analysis populations**

| **Analysis step** | **Remaining N** | **Excluded at step, n** | **Reason or analytic role** |
| --- | --- | --- | --- |
| Initial treated cohort | 286 |  | Source real-world cohort. |
| Baseline AFP >=20 ng/mL | 218 | 68 | AFP-non-evaluable for AFP-response phenotype. |
| Day 28-70 laboratory-complete phenotype evaluable cohort | 185 | 33 | Missing or outside-window day 28-70 laboratory values among AFP-eligible patients. |
| Primary day-70 analytic cohort | 166 | 19 | Not event-free or not under follow-up at day 70 among phenotype-evaluable patients. |
| Joint phenotype-imaging cohort | 165 | 1 | Missing or non-evaluable first RECIST imaging among the primary day-70 cohort. |
| RECIST stable-disease joint cohort | 79 |  | Nested exploratory subgroup within the joint phenotype-imaging cohort. |
| mRECIST stable-disease joint cohort | 59 |  | Nested exploratory subgroup within the joint phenotype-imaging cohort. |

RECIST and mRECIST stable-disease cohorts were nested exploratory populations within the joint phenotype-imaging cohort and were not part of the primary day-70 survival analysis.

**Supplementary Table S3. Selected characteristics of included and excluded source-cohort patients**

| **Characteristic** | **Included day-70 cohort (n=166)** | **Excluded / non-analytic patients (n=120)** |
| --- | --- | --- |
| Age, years | 58.4 (52.4-64.5) | 58.4 (51.9-65.3) |
| Male sex | 144/166 (86.7%) | 101/120 (84.2%) |
| ECOG 0 | 41/166 (24.7%) | 35/120 (29.2%) |
| ECOG 1 | 107/166 (64.5%) | 69/120 (57.5%) |
| ECOG 2 | 18/166 (10.8%) | 16/120 (13.3%) |
| BCLC stage C | 95/166 (57.2%) | 64/120 (53.3%) |
| Macrovascular invasion | 87/166 (52.4%) | 48/120 (40.0%) |
| Extrahepatic metastasis | 60/166 (36.1%) | 32/120 (26.7%) |
| Tumor burden score | 10.3 (7.9-13.3) | 8.3 (6.2-10.8) |
| Child-Pugh class B | 87/166 (52.4%) | 56/120 (46.7%) |
| Baseline ALBI score | -2.2 (-2.5--2.0) | -2.2 (-2.4--1.9) |
| HBV-related etiology | 130/166 (78.3%) | 96/120 (80.0%) |
| Baseline AFP, ng/mL | 747.6 (263.2-2611.8) | 13.9 (6.6-599.2) |
| ICI + TKI | 85/166 (51.2%) | 64/120 (53.3%) |
| ICI + anti-VEGF antibody | 69/166 (41.6%) | 42/120 (35.0%) |
| Dual ICI / other ICI combination | 12/166 (7.2%) | 14/120 (11.7%) |
| Early local therapy within 8 weeks | 42/166 (25.3%) | 31/120 (25.8%) |
| Follow-up from treatment initiation, months | 16.1 (10.2-24.2) | 13.7 (8.0-21.8) |
| Progression or death observed from treatment initiation | 130/166 (78.3%) | 104/120 (86.7%) |
| Death observed from treatment initiation | 64/166 (38.6%) | 72/120 (60.0%) |

Values summarize selected source-cohort differences after applying AFP-evaluable and day-70 landmark requirements.

**Supplementary Table S4. Missingness, structural non-applicability, and analysis ineligibility**

| **Category** | **Variable or condition** | **N** | **Percent** | **Reason** |
| --- | --- | --- | --- | --- |
| True missingness | Landmark AFP | 40 | 14.0 | Missing early AFP value in the source cohort. |
| True missingness | Landmark albumin | 40 | 14.0 | Missing early albumin value in the source cohort. |
| True missingness | Landmark total bilirubin | 40 | 14.0 | Missing early bilirubin value in the source cohort. |
| True missingness | Landmark laboratory date | 40 | 14.0 | Missing date for early laboratory assessment. |
| True missingness | First imaging date | 27 | 9.4 | Missing first post-treatment imaging date. |
| True missingness | RECIST response | 27 | 9.4 | Missing or non-evaluable RECIST response. |
| True missingness | mRECIST response | 27 | 9.4 | Missing or non-evaluable mRECIST response. |
| Structural not applicable | Death date | 150 |  | Alive or death not observed by last follow-up. |
| Structural not applicable | Progression date | 52 |  | Progression not observed and censored. |
| Structural not applicable | Second-line treatment start date | 229 |  | No second-line treatment recorded. |
| Structural not applicable | AFP-ALBI phenotype | 68 |  | Baseline AFP <20 ng/mL; AFP-response phenotype not applicable. |
| Analysis ineligible | Baseline AFP <20 ng/mL | 68 |  | Excluded from AFP-evaluable phenotype analysis. |
| Analysis ineligible | Day 28-70 phenotype not evaluable | 33 |  | Incomplete or outside-window phenotype assessment. |
| Analysis ineligible | Not event-free or not followed at day 70 | 19 |  | Excluded from primary day-70 landmark analysis. |
| Analysis ineligible | No first RECIST imaging for joint cohort | 1 |  | Excluded from joint phenotype-imaging cohort. |

Death date, progression date, and second-line treatment date were not treated as ordinary missingness when the event or treatment was not observed. Baseline AFP <20 ng/mL was treated as structural non-applicability for the AFP-response phenotype.

**Supplementary Table S5. RECIST inter-reader agreement**

| **Reader 1 / Reader 2 or summary item** | **CR** | **PR** | **SD** | **PD** |
| --- | --- | --- | --- | --- |
| **Agreement matrix** | | | | |
| CR | 2 | 1 | 0 | 0 |
| PR | 1 | 51 | 6 | 0 |
| SD | 0 | 2 | 108 | 17 |
| PD | 0 | 0 | 6 | 65 |
| **Summary estimates** | | | | |
| Paired readings, n | 259 |  |  |  |
| Observed agreement | 0.873 |  |  |  |
| Unweighted Cohen kappa | 0.801 |  |  |  |
| 95% CI for kappa | 0.735-0.858 |  |  |  |
| Method | Bootstrap percentile CI with 500 resamples |  |  |  |

The agreement matrix summarizes Reader 1 versus Reader 2 categories. Summary estimates are shown below the matrix. CR, complete response; PD, progressive disease; PR, partial response; RECIST, Response Evaluation Criteria in Solid Tumors; SD, stable disease.

**Supplementary Table S6. Cross-classification under alternative phenotype definitions**

| **Phenotype definition** | **Concordant favorable** | **Response-liver vulnerability** | **Nonresponse-liver preserved** | **Concordant failure** |
| --- | --- | --- | --- | --- |
| Primary modified ALBI phenotype | 58 | 36 | 43 | 29 |
| AFP response cutoff >=30% | 51 | 30 | 50 | 35 |
| AFP response cutoff >=50% | 31 | 18 | 70 | 47 |
| Standard ALBI-grade deterioration | 66 | 28 | 48 | 24 |

Counts represent phenotype sizes under each alternative AFP or ALBI definition. The primary definition used an AFP decline of at least 20% and modified ALBI deterioration.

**Supplementary Table S7. Sensitivity analyses for progression-free survival**

| **Sensitivity analysis** | **N/events** | **HR (95% CI)** | **P value** |
| --- | --- | --- | --- |
| Primary day-70 landmark | 166/130 | 1.79 (1.18-2.72) | 0.006 |
| Day-42 restricted laboratory window | 79/65 | 2.63 (1.32-5.21) | 0.006 |
| Actual laboratory-date landmark | 179/143 | 1.78 (1.19-2.65) | 0.005 |
| Excluding Child-Pugh class B | 79/61 | 1.92 (1.03-3.59) | 0.041 |
| Excluding early local therapy within 8 weeks | 124/100 | 1.68 (1.06-2.65) | 0.027 |
| AFP response cutoff >= 30% | 166/130 | 1.72 (1.12-2.66) | 0.013 |
| AFP response cutoff >= 50% | 166/130 | 2.20 (1.31-3.70) | 0.003 |
| Standard ALBI-grade deterioration | 166/130 | 1.65 (1.09-2.48) | 0.017 |

Effect estimates compare non-favorable phenotype with concordant favorable phenotype. PFS models used the same covariate structure as the primary binary PFS model when estimable. CI, confidence interval; HR, hazard ratio.

**Supplementary Table S8. Sensitivity analyses for overall survival**

| **Sensitivity analysis** | **N/events** | **HR (95% CI)** | **P value** |
| --- | --- | --- | --- |
| Primary day-70 landmark | 166/64 | 1.23 (0.68-2.22) | 0.503 |
| Day-42 restricted laboratory window | 79/32 | 1.29 (0.50-3.32) | 0.603 |
| Actual laboratory-date landmark | 179/73 | 1.24 (0.71-2.17) | 0.449 |
| Excluding Child-Pugh class B | 79/25 | 1.09 (0.42-2.84) | 0.863 |
| Excluding early local therapy within 8 weeks | 124/53 | 1.18 (0.63-2.20) | 0.606 |
| AFP response cutoff >= 30% | 166/64 | 1.22 (0.65-2.28) | 0.534 |
| AFP response cutoff >= 50% | 166/64 | 2.37 (1.00-5.61) | 0.049 |
| Standard ALBI-grade deterioration | 166/64 | 1.07 (0.60-1.92) | 0.810 |

Effect estimates compare non-favorable phenotype with concordant favorable phenotype using reduced adjusted Cox models. OS sensitivity estimates were exploratory because deaths were fewer. CI, confidence interval; HR, hazard ratio.

**Supplementary Table S9. Subgroup analyses for progression-free survival**

| **Subgroup** | **Level** | **Favorable events/N** | **Non-favorable events/N** | **Adjusted HR (95% CI)** | **P value** | **P for interaction** |
| --- | --- | --- | --- | --- | --- | --- |
| BCLC stage | B | 18/28 (64.3%) | 34/43 (79.1%) | 1.49 (0.82-2.73) | 0.193 | 0.408 |
|  | C | 19/30 (63.3%) | 59/65 (90.8%) | 2.08 (1.20-3.63) | 0.009 |  |
| Child-Pugh class | A | 20/32 (62.5%) | 41/47 (87.2%) | 1.76 (0.99-3.14) | 0.054 | 0.966 |
|  | B | 17/26 (65.4%) | 52/61 (85.2%) | 1.79 (1.01-3.18) | 0.046 |  |
| Macrovascular invasion | No | 21/33 (63.6%) | 39/46 (84.8%) | 2.10 (1.19-3.71) | 0.011 | 0.407 |
|  | Yes | 16/25 (64.0%) | 54/62 (87.1%) | 1.49 (0.83-2.68) | 0.177 |  |
| Extrahepatic metastasis | No | 25/39 (64.1%) | 59/67 (88.1%) | 2.00 (1.20-3.34) | 0.008 | 0.434 |
|  | Yes | 12/19 (63.2%) | 34/41 (82.9%) | 1.44 (0.73-2.83) | 0.295 |  |
| HBV-related etiology | No | 10/14 (71.4%) | 19/22 (86.4%) | 1.37 (0.62-3.05) | 0.438 | 0.452 |
|  | Yes | 27/44 (61.4%) | 74/86 (86.0%) | 1.96 (1.20-3.20) | 0.007 |  |
| Prior local therapy | No | 21/33 (63.6%) | 62/72 (86.1%) | 1.43 (0.84-2.41) | 0.186 | 0.236 |
|  | Yes | 16/25 (64.0%) | 31/36 (86.1%) | 2.33 (1.22-4.44) | 0.010 |  |

The exposure was non-favorable phenotype versus concordant favorable. P for interaction was obtained from a multiplicative interaction term and is shown once for each subgroup category. These analyses were exploratory.

Supplementary Table S10. Systemic regimen distribution and exploratory interaction context

| **Systemic regimen class** | **N** | **PFS events** | **Concordant favorable, n** | **Non-favorable, n** | **Comment** |
| --- | --- | --- | --- | --- | --- |
| Dual ICI / other ICI combination | 12 | 11 | 4 | 8 | Sparse; excluded from two-major-class interaction model |
| ICI + TKI | 85 | 70 | 29 | 56 | Adjusted non-favorable HR 1.74 (95% CI 1.01-3.00) |
| ICI + anti-VEGF antibody | 69 | 49 | 25 | 44 | Adjusted non-favorable HR 2.45 (95% CI 1.27-4.72) |

The interaction analysis was restricted to ICI + TKI and ICI + anti-VEGF antibody (n=154; 119 PFS events). P for interaction=0.422. The dual-ICI/other-combination group was too small for stable effect-modification estimates.

**Supplementary Table S11. Stable-disease joint cohorts**

| **Stable-disease cohort** | **N** | **PFS events** | **OS events** | **Concordant favorable, n** | **Non-favorable, n** | **Interpretation** |
| --- | --- | --- | --- | --- | --- | --- |
| RECIST stable disease | 79 | 61 | 24 | 30 | 49 | Exploratory joint-landmark subgroup |
| mRECIST stable disease | 59 | 42 | 13 | 23 | 36 | Exploratory joint-landmark subgroup |

Stable-disease analyses used the joint phenotype-imaging landmark and were interpreted as exploratory monitoring analyses rather than treatment-decision rules.

**Supplementary Table S12. Robustness analyses by treatment class, continuous ALBI change, baseline liver reserve, and cause of death**

| **Analysis** | **Contrast / quantity** | **N/events** | **Effect estimate (95% CI)** | **P value** | **P for interaction** |
| --- | --- | --- | --- | --- | --- |
| Systemic regimen | Non-favorable within ICI + anti-VEGF antibody | 69/49 | HR 2.45 (1.27-4.72) | 0.008 | 0.422 |
| Systemic regimen | Non-favorable within ICI + TKI | 85/70 | HR 1.74 (1.01-3.00) | 0.045 |  |
| Continuous ALBI model | AFP response | 166/130 | HR 0.57 (0.39-0.83) | 0.003 |  |
| Continuous ALBI model | ALBI increase per 0.5 unit | 166/130 | HR 1.33 (1.04-1.68) | 0.021 |  |
| Continuous ALBI interaction | AFP response x ALBI change | 166/130 | Interaction HR 1.05 (0.67-1.64) | 0.828 | 0.828 |
| Baseline modified ALBI | Non-favorable within grades 1/2a | 75/51 | HR 1.73 (0.96-3.13) | 0.070 | 0.814 |
| Baseline modified ALBI | Non-favorable within grades 2b/3 | 91/79 | HR 1.90 (1.09-3.31) | 0.023 |  |
| Cause-specific mortality | HCC-progression death | 166/40 | HR 1.39 (0.69-2.78) | 0.354 |  |
| Cause-specific mortality | Liver-failure death | 166/15 | HR 1.49 (0.47-4.67) | 0.498 |  |

Regimen and baseline-liver-reserve interactions were exploratory. Cause-specific models treated deaths from other causes as competing events censored at the time of death. CI, confidence interval; HR, hazard ratio; ICI, immune checkpoint inhibitor; TKI, tyrosine kinase inhibitor; VEGF, vascular endothelial growth factor.

**Supplementary Figure Legends**

**Supplementary Figure S1. True missingness profile.** The figure displays missing early laboratory and imaging fields after separating structural non-applicability and analysis ineligibility.

**Supplementary Figure S2. Sensitivity analyses for progression-free survival.** Estimates compare non-favorable phenotype with concordant favorable phenotype using adjusted Cox models. CI, confidence interval; HR, hazard ratio.

**Supplementary Figure S3. Subgroup analyses for progression-free survival.** The table-style forest plot shows events/N, adjusted hazard ratios, 95% confidence intervals, P values, and one P for interaction per subgroup category. The vertical dashed line indicates HR=1. CI, confidence interval; HR, hazard ratio.

**Supplementary Figure S4. Exploratory sensitivity analyses for overall survival.** Estimates compare non-favorable phenotype with concordant favorable phenotype using reduced adjusted Cox models. CI, confidence interval; HR, hazard ratio.

**Supplementary Figure S5. Phenotype distribution among patients with mRECIST stable disease.** This nested joint-landmark analysis was exploratory. mRECIST, modified Response Evaluation Criteria in Solid Tumors; SD, stable disease.

Supplementary Figure S6. Proportional hazards diagnostics. Bars show term-level P values from Schoenfeld-residual diagnostics for the revised primary PFS and reduced OS Cox models. The vertical reference line is P=0.05; values below this threshold suggest possible non-proportionality and require cautious interpretation.
